# Comparative Analyses of Response Surface Methodology and Artificial Neural Network on Medium Optimization for *Tetraselmis* sp. FTC209 Grown under Mixotrophic Condition

**DOI:** 10.1155/2013/948940

**Published:** 2013-09-10

**Authors:** Mohd Shamzi Mohamed, Joo Shun Tan, Rosfarizan Mohamad, Mohd Noriznan Mokhtar, Arbakariya B. Ariff

**Affiliations:** ^1^Department of Bioprocess Technology, Faculty of Biotechnology and Biomolecular Sciences, Universiti Putra Malaysia, 43400 Serdang, Selangor, Malaysia; ^2^Institute of Bioscience, Universiti Putra Malaysia, 43400 Serdang, Selangor, Malaysia; ^3^Department of Process and Food Engineering, Faculty of Engineering, Universiti Putra Malaysia, 43400 Serdang, Selangor, Malaysia

## Abstract

Mixotrophic metabolism was evaluated as an option to augment the growth and lipid production of marine microalga *Tetraselmis* sp. FTC 209. In this study, a five-level three-factor central composite design (CCD) was implemented in order to enrich the W-30 algal growth medium. Response surface methodology (RSM) was employed to model the effect of three medium variables, that is, glucose (organic C source), NaNO_3_ (primary N source), and yeast extract (supplementary N, amino acids, and vitamins) on biomass concentration, *X*_max_, and lipid yield, *P*_max_/*X*_max_. RSM capability was also weighed against an artificial neural network (ANN) approach for predicting a composition that would result in maximum lipid productivity, Pr_lipid_. A quadratic regression from RSM and a Levenberg-Marquardt trained ANN network composed of 10 hidden neurons eventually produced comparable results, albeit ANN formulation was observed to yield higher values of response outputs. Finalized glucose (24.05 g/L), NaNO_3_ (4.70 g/L), and yeast extract (0.93 g/L) concentration, affected an increase of *X*_max_ to 12.38 g/L and lipid a accumulation of 195.77 mg/g dcw. This contributed to a lipid productivity of 173.11 mg/L per day in the course of two-week cultivation.

## 1. Introduction

The microalga *Tetraselmis *has been used extensively in aquaculture, especially for rearing larval stages of mollusks and crustaceans [1]. In light of favorable outlooks [2], an emerging application of *Tetraselmis* or other oleaginous microalgae is currently centered on carbon biofixation in tandem with bioconversion towards renewable fuels. The whole process is considered to be efficient and encouragingly leaves a small pollution footprint [3]. Several species of *Tetraselmis* are acknowledged to possess metabolic plasticity, whereby in response to the culture conditions, these species can manifest in alternative phenotypes resulting in altered formation of algal bioproducts [4].

Heterotrophy of exogenous nutrients by microalgae is now being regarded as the practical means to increase the volumetric productivity of algal biomass [5]. Nonetheless, the amount of neutral lipid, a principal component in biodiesel synthesis, was significantly diminished for *Tetraselmis* cultured with organic carbon substrates without illumination. For an unspecified species of *Tetraselmis*, Day and Tsavalos [6] found that cultivation with glucose yielded only 0.64% *w/w* cellular lipid obtained under complete darkness, as opposed to 3.71% *w/w* for culture exposed to light. A study by Azma et al. [1] on the heterotrophy of *T. suecica* has reported a respectable 28.8 g/L dried cell weight. Lipid productivity was claimed to increase by about 2 times. However, comparison was made solely against photoautotrophic culture, by which the typical biomass concentration is in the range of 0.1 to 1.0 g/L, owing to the effect of mutual shading [7]. Alternatively, researchers are looking into the potential of mixotrophic (photoheterotrophic) mode upon realization of enhanced growth and unrepressed light-dependent bioproducts formation from the combined effects of photosynthesis and the cells' own ability to ingest either prey or dissolved organic materials [8].

Mixotrophy in the commercial-scale open ponds of *Chlorella* and *Spirulina* has been practiced for some time through continuous addition of acetate in small quantities during daytime to support greater growth [9]. Zhao et al. [10] had found that the mode was conducive for both biomass and lipid accumulations of *Scenedesmus quadricauda*, wherein *X*_max⁡_ and lipid, *P*_max⁡_, were registered at 3.36 g/L and 0.79 g/L, respectively when culture was fed with starch wastewater. In addition, Isleten-Hosoglu et al. [11] also proposed that the mixotrophicallygrown *Ettlia texensis *isto be a good biofuel-producer candidate, where imposing optimized mixing and medium in stirred tank environment would lead to *X*_max⁡_ of 10.1 g/L, with microalga retaining 35% of lipid bodies during its 11 days of cultivation, corresponding to biomass and lipid productivities of 0.92 g/L per day and 322 mg/L per day, respectively.

Previous observations on the native isolate *Tetraselmis* sp. FTC 209 in our laboratory revealed the strain preference towards mixotrophic over other forms of metabolisms. Initial attempt on medium design was based on the concept of elemental balance of aqueous nutrients to match the stoichiometry of the particular algal species [12]. The technique comprises a straightforward increase of nutrients found lacking to promote a higher cell-density culture, but neglecting the combined interactions between medium components involved. In a way, it may not guarantee to pinpoint the exact optimal condition, which possibly leads to slight inaccurate conclusions [13]. Statistical methods have been applied for developing reliable culture system. Of late, response surface methodology (RSM) coupled with central composite design (CCD) has been a popular tool to model the probable curvature of the measured responses in algal medium formulation [1, 11, 13, 14]. However, a major obstacle in reaching model accuracy and generalization lies in the nonlinearity and time-varying nature of bioprocesses [15]. Artificial neural network (ANN) has been progressively applied in a number of optimization works [16]. ANN is a highly interconnected network of processing elements (neurons) capable of massive parallel computations, representing a data-centric modeling inspired by biological nervous system. Contrary to the conventional model requiring that the order needs to be stated (i.e., second, third or fourth order), ANN is more flexible and does not impose any restriction on the type of relationship governing the dependence output parameters on the various running conditions [17]. ANN essentially transforms inputs that passed through network of neurons with weighted interconnection into outputs predicted to the best of its ability. It is adaptive or trainable with a given dataset via adjusting many network factors (no. of layers, no. of neurons in hidden layers, types of transfer functions, or learning algorithms). The process continues until a defined accuracy has been reached [18].

Linear regression modeling via RSM has been constructed in the past for *T. suecica *[1]. However, no study to date has made use of ANN capacity for simulating the structure and functional aspects of neural networks to precisely develop an optimal growth medium for *Tetraselmis*. The aim of this study was to analyze the potential improvement of predictive microbiology afforded by RSM and ANN-based models, in this case, by assessing the contribution of major organic carbon and nitrogen sources such as glucose, NaNO_3_, and yeast extract towards enhancing the lipid productivity. 

## 2. Materials and Methods

### 2.1. Microalga Strain, Maintenance, and Inoculum Preparation

The microalga, *Tetraselmis* sp. FTC 209 previously isolated from the coastal waters of Port Dickson, Negeri Sembilan (Malaysia), was obtained from the collection of Fermentation Technology Unit (FTU-GMP@BIOTECH) of University Putra Malaysia. Prior to microalgal characterization via morphological, 16S rDNA partial gene sequencing and phylogenetic analysis had disclosed a taxonomic position of the strain as closely related to *Tetraselmis striata*. The axenicity of *Tetraselmis* isolate was maintained by culturing the cells onto Walne's medium agar treated with antibiotics cocktail comprising of 100/25 mg/L of ampicillin/streptomycin and fortified with 5 g/L glucose.

Four-week old colonies grown on agar plates were collected with disposable loops and cultivated in liquid medium formulated beforehand according to the principle of elemental balance. In brief, the technique requires increasing the concentrations of any bioelements found deficient in the standard Walne's medium by matching to the alga's cellular elemental composition. Nonetheless, a drop in *Tetraselmis *sp. FTC 209 growth rate and cell density was observed if such increment, expressed in “percent biomass capacity” for all macronutrients that were raised higher than 30%. The modified basal medium, designated as W-30 used throughout this study, was formulated using sterile double-filtered seawater and composed of (g/L): (NH_4_)_2_SO_4_, 0.377; KH_2_PO_4_, 0.362; K_2_HPO_4_, 0.200; MgSO_4_·7H_2_O, 0.144; CaCl_2_·2H_2_O, 0.327; FeCl_3_·6H_2_O, 0.0091; MnCl_2_·4H_2_O, 0.0044; Na_2_EDTA·2H_2_O, 0.200; H_3_BO_3_, 0.336; C_6_H_8_O_7_, 0.020; ZnCl_2_, 0.0021; CuSO_4_·5H_2_O, 0.002; CoCl_2_·6H_2_O, 0.002; Na_2_MoO_4_·2H_2_O, 5.0 × 10^−4^; thiamine-HCl, 2.0 × 10^−5^; cyanocobalamine, 1.0 × 10^−5^, and biotin, 2 × 10^−7^. The microalga was serially subcultured by aseptically transferring 10% (*v/v*) of culture into fresh liquid medium at two-week intervals. Cultures having the cell number of approximately 32 × 10^6^ cells/mL were used as a standard inoculum in all the following optimization experiments.

### 2.2. Mixotrophic Experiments

All mixotrophic cultivation experiments of *Tetraselmis* sp. FTC 209 were performed in 500 mL Erlenmeyer flasks containing 200 mL of liquid medium. The sterile flasks were inoculated with 20 mL inoculum and incubated in an orbital shaker (Ecotron, Infors-HT, Switzerland) at 27°C with agitation fixed at 130 rpm. Light source was provided through manual installation of T5 fluorescent tubes (OSRAM, Germany) inside the shaker unit, illuminating the cultures at about 2280 ± 250 lux or an equivalent photosynthetic photon flux (PPF) of 30 ± 3.3 *μ*mol/m^2^·s for a constant diurnal cycle of 12 h (light) : 12 h (dark). All runs were carried out in triplicates for a total duration of 2 weeks cultivation.

### 2.3. Experimental Design

Based on prior mixotrophic cultivations with W-30 medium, *X*_max⁡_ was recorded at 8.08 g/L with lipid embodied about 20–22% of the *Tetraselmis* cell after 500 h of cultivation, by having the presence of glucose in the range of 20–30 g/L, and NaNO_3_ being supplied at 3.50–5.23 g/L, respectively. Addition of organic complex nutrients, for example, peptone, yeast extract, malt extract, or beef extract to further boost the biomass propagation, and therefore indirectly, the lipid yield, have been suggested in the reports pertaining to mixotrophy or heterotrophy of several *Tetraselmis *species [1, 4, 19]. The foremost among the proposed supplements is yeast extract, by and large sourced from the autolysate of spent *Saccharomyces* cells, an underutilized waste by-product of brewing industry [20]. It has a competitively lower price compared to other organic N sources [11] and generally the most preferred for production-scale bioreactor [5]. 

A five-level, full-factorial central composite design (CCD) with three independent variables, that is, the concentrations of glucose (Merck Co.), yeast extract (Merck Co.), and NaNO_3_ (Kollins Chemicals) was applied in this study (Table 1), requiring 19 sets of experimental runs consisting of 8 factorial (cubic points), 6 axial (star points), and 5 replicates of center points. The effects of these medium constituents towards ultimately achieving the maximum lipid productivity, Pr_lipid_ (mg/L per day), were identified. Subsequent experimental values acquired from the runs using predicted optimal conditions were then used as validating set and were compared with the computed optimal values. 

### 2.4. Response Surface Methodology Modeling

RSM was employed to optimize the cultivation plus to investigate the relative and interactive effects the three medium constituents. To comprehend the algal growth and lipid secretion behavior, three responses comprising of biomass concentration (g/L), lipid yield (mg lipid/g dcw), and lipid productivity (mg/L per day) were initially measured. Design Expert (version 7.1.6, Stat-Ease Inc., Minneapolis, MN, USA) was used for regression modeling and data interpretation. The observed responses from CCD design were then fitted to the following polynomial equation as shown by

(1) $$\begin{array}{c} Y=\beta_{0}+\overset{k}{\underset{i=1}{\sum }}\beta_{i}x_{i}+\overset{k}{\underset{i=1}{\sum }}\beta_{ii}x_{i}^{2}+\overset{k}{\underset{i=1}{\sum }}\overset{k}{\underset{j=1}{\sum }}\beta_{ij}x_{i}\beta_{ii}x_{j}+\varepsilon , \end{array}$$

where *Y* is the predicted response; *i* and *j* are the index numbers for the pattern; *β* is the offset term; *β*_*i*_, *β*_*ii*_, and *β*_*ij*_ are the coefficients for the linear, quadratic, and interaction effects, respectively; *x*_*i*_ and *x*_*j*_ are the coded variables; and *ε* is the error. The regression equation was optimized by an iterative method to achieve the optimum values. 

### 2.5. Artificial Neural Network Modeling

NeuralPower (version 2.5, CPC-X Software, USA) is a powerful ANN module for forecasting nonlinear regression. It was chosen to conduct pattern recognition on similar dataset subjected to RSM analysis. Data were divided into two sets; training set (15 data) and testing set (4 data) which was randomly picked from Table 2 (bold numbers). Every network possesses three input variables and one output response, each underwent training for computation of network parameters. Network performance was simultaneously consulted with the testing set during training to avoid becoming “over trained” and thereby improves the prediction (i.e., generalization) towards any data excluded from the training sets [21]. In the event of supervised training, designed networks were trained to the point of exhibiting root mean square error (RMSE) as shown by (1) to be as closest to 0.01, whereas the networks' correlation coefficient (*R*) and determination coefficient (DC) as defined by (2) and (3), respectively, are closest or equal to 1:

(2) $$\begin{array}{c} \text{RMSE}={\left[\frac{{\sum_{i=1}^{N}\left(x_{p}-x_{\text{obs}}\right)}^{2}}{N}\right]}^{1/2}, \end{array}$$

(3) $$\begin{array}{c} R^{}=\frac{\sum_{i=1}^{N}\left(x_{\text{obs}}-x_{m}\right)\left(x_{p}-x_{pm}\right)}{\sqrt{{\sum_{i=1}^{N}\left(x_{\text{obs}}-x_{m}\right)}^{2}}\sqrt{{\sum_{i=1}^{N}\left(x_{p}-x_{pm}\right)}^{2}}}, \end{array}$$

(4) $$\begin{array}{c} \text{DC}=1-\frac{\sum_{i=1}^{N}{\left(x_{p}-x_{\text{obs}}\right)}^{2}}{{\sum_{i=1}^{N}\left(x_{\text{obs}}-x_{m}\right)}^{2}}, \end{array}$$

where *N* is the number of data points, *x*_obs_ is the observed value, *x*_*p*_ is the predicted value obtained from ANN model, *x*_*m*_ is the average of actual values, and *x*_*pm*_ is the average of predicted values. 

A full feed-forward network structure was selected for modeling of lipid productivity. In this case, network comprises of three input neurons, one output (response) neuron, and a single hidden layer, which is highly recommended for most practical feed-forward network designs [18]. Networks were consecutively trained via different learning algorithms (the “standard” back propagation package; genetic algorithm, GA; and Levenberg-Marquardt, LM). Adjustment of network parameters encompassed on the number of neurons in hidden layer and the types of transfer functions for both hidden and output layers. The trial and error approach was sufficient in choosing the optimal number of neurons that translates to the best network topology [22]. In NeuralPower; the numbers were tested from 5 to 30, each with the increment of one neuron at a time. The common transfer functions used for nonlinear regression are sigmoid, hyperbolic tangent, and Gaussian [23]. Linearity of network was also tested using linear, bipolar linear, and threshold linear-types of transfer functions. The search for optimal network topology proceeded by iteratively developing several networks. Each would be trained to meet the acceptable residual error terms as stipulated by (2) to (4). Other parameters such as the learning rate and momentum coefficient were kept to the default values of the software. 

### 2.6. Verification of Predicted Data

The estimation capabilities of both RSM and ANN models were evaluated by means of comparing the responses computed from both methods to the observed data. The calculated coefficients of determinations, *R* ^2^ or DC (4), were exploited for the purpose of comparison, whether to determine the most accurate ANN model amongst various generated topologies, and from such outcome, the aforementioned best model would be compared to RSM results. *R* ^2^ represents the proportion of the total sample variability as explained by the given regression. Nonetheless, it is not a sole measurement of model accuracy. The use of RMSE (2) or an absolute relative error test is more appropriate to describe the deviations. Apart from *R* ^2^, RMSE and mean absolute error (MAE) as defined by (5) were chosen as ancillary statistical indicators to measure the model performance

(5) $$\begin{array}{c} \text{MAE}=\frac{1}{N}\overset{N}{\underset{i=1}{\sum }}\left|x_{\text{obs}}-x_{p}\right|. \end{array}$$

Model is considered accurate when *R* ^2^ is closest to 1.0, while RMSE and MAE between predicted and observed data must be as small as possible. Acceptable values of *R* ^2^, RMSE, and MAE mean that model equation is able to describe the true behaviour of the system, and it can be applied for interpolation in the experimental domain [16].

### 2.7. Analytical Methods

*Tetraselmis* biomass concentration (g/L) was determined gravimetrically after the cells were lyophilized overnight in a preweighed sample vials. Culture samples of known volume were washed beforehand with 0.5 M ammonium formate to remove excess sea salts, followed by at least twice with distilled water. The resuspended cell pellets following recentrifugation at 2465 rcf for 5 min (5810 R, Eppendorf, Germany) were later subjected to freeze-drying. The procedure consists of prefreezing (−30°C) at 1 bar for 4 h, sample preparation for 15 min at 1 bar, main drying (30°C) for 20 h at 0.001 mbar, and concluded with final drying at 0.0001 mbar for 15 min (Epsilon 1-8D, Martin Christ, Germany). 

Lipid was extracted from 300 to 400 mg of dried cells according to a method modified from Folch et al. [24]. Cells were first pulverized to fine granules by pestle and mortar, followed by adding 4 mL methanol containing 500 ppm butylated hydroxytoluene (BHT), 2 mL chloroform, and 0.4 mL water. The mixture was homogenized and disrupted in an ultrasonic bath (Thermo-10D, Thermoline, Australia) for 15 min. Additional chloroform (2 mL) was added, and the mixture was left standing for one day. Water (2 mL) was later added, and the mixture was vortexed for 60 s. After centrifugation and siphoning off the upper phase, the lower chloroform phase containing lipid was collected in a pre-weighed vial. Organic solvent was heated to 62°C and purged with a passing nitrogen stream. The total lipid was also determined gravimetrically. 

Qualitative inspection of intracellular lipid was also conducted using fluorescent microscopy. Nile red (9-(diethylamino)-5H-benzo [a] phenoxazin-5-one), a photostable, selective fluorescent dye, was used for *in situ* staining of neutral lipid. 30 *μ*L of algal cells suspensions sampled at the end of cultivation as mixed with 10 *μ*L of 0.1 mg/mL Nile red solution (Sigma) (dissolved in acetone). 1960 *μ*L of a freshly prepared 25% (*v/v*) dimethylsulfoxide (DMSO) solvent was then added as stain carrier since the thick, rigid cell walls of *Tetraselmis* sp. could inhibit the permeation of fluorescent dye [25]. The mixture was vortexed for about 60 s and incubated in the dark at 40°C for 10 min. Microalga cells were photographed using light microscope (Leica DMLB, Wetzlar GmbH, Germany) with an eye-piece digital camera (Dino-Eye AM4023X, ANMO Electronics, Taiwan). Epifluorescent images of Nile red stained cells were captured using D filter cube (broad-range UV+ violet excitation) or N2.1 filter cube (green excitation) obtained at 1000x magnification with oil immersion (Leica Microsystems).

## 3. Results and Discussion

### 3.1. RSM Modeling

Table 2 displays the CCD design matrix of the medium constituents chosen, together with the actual responses, that is, biomass concentration, lipid yield, and productivity. Sequential comparison of all the potential RSM models' sum of squares by Design Expert software has demonstrated that the quadratic type is the highest order polynomial regression aptly suitable to explain the relationship between input variables and responses. The corresponding uncoded second-order polynomial response equations derived accordingly for the algal biomass (6), lipid yield (7), and lipid productivity (8) are as follow:

(6) Xmax⁡=−20.02+0.85x1+6.26x2+4.50x3−0.015x12   −1.00x22−0.50x32+0.025x1x2   +0.052x1x3−0.375x2x3,

(7) Pmax⁡Xmax⁡=−1018.70+65.73x1+245.59x2+107.03x3 −1.20x12−91.53x22−7.74x32 −1.49x1x2−1.19x1x3−0.60x2x3,

(8) Prlipid=−1188.07+69.87x1+257.95x2+128.27x3 −1.31x12−89.96x22−11.07C2   −1.25x1x2−0.49x1x3−3.17x2x3.

The goodness of fit of each equation is denoted by *R* ^2^_Adj_. Model assessment that was based on *R* ^2^_Adj_ in place of *R* ^2^ was more accurate, given that the presence of extraneous factorial terms in a derived model equation will result in some reduction in the error sum of squares. *R* ^2^_Adj_ will compensate for the added explanatory variables since *R* ^2^ value naturally increases with the addition of new variable terms. *R* ^2^_Adj_ in this case are 0.868, 0.914, and 0.970 for (6) to (8), respectively, indicating good model agreement between the observed against predicted values for all the output responses.

**Figure 1:**
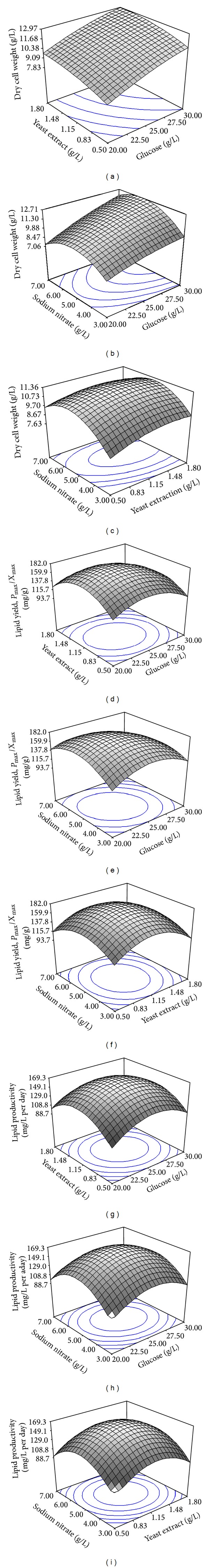
Surface response plots of (a, b, c) biomass concentration, (d, e, f) intracellular lipid, and (g, h, i) overall lipid productivity as modeled via RSM.

Statistical testing for significances of the proposed models is presented by the analysis of variance (ANOVA) in Table 3. According to the results, the individual *R* ^2^ obtained at 0.934, 0.957, and 0.985 shows that the three derived models could explain more than 93% of the variability. The *F*-test value of 14.20 for biomass concentration, 22.14 for lipid yield, and 64.82 for lipid productivity, plus the probability values (*P*_model_ > *F*) of less than 0.05, indicates that each of these models were considered significant. Besides, relative variability of the experimental results was confirmed to be acceptable based on the individual coefficient of variation (CV) for biomass (10.30%), lipid yield (12.72%), and productivity (10.51%). Another cue for the goodness of fit is represented by the models' lack of fit (LOF) terms which were proven to be insignificant, whereby (*P*_model_ > *F*) were determined at 0.1907, 0.5391, and 0.4416, respectively. The optimal conditions and interactions between the medium constituents are shown in the three dimensional response surface plots (Figure 1). The biomass concentration was varied from 4.95 (g/L) to 13.05 (g/L). On the other hand, the intracellular lipid yield range was varied from 58.87 (mg lipid/g dcw) to 204.30 (mg lipid/g dcw), while its related productivity was varied from 31.72 (mg/L per day) to 177.20 (mg/L per day). 

As per ANOVA analysis, all three independent input variables directly contributed to the first-order effect on the cell growth model. However, the quadratic effect of NaNO_3_ (*x*_3_^2^) was more prominent (*P* < 0.0001) compared to the other inputs. By maintaining the NANO_3_ concentration at its center point, the cell density was observed as increasing in an almost linear fashion with the increase in both glucose and yeast extracts (Figure 1(a)). From the examination of contour plots, the highest biomass concentration was obtained with glucose ranging from 26.0 to 30 g/L, and yeast extract from 0.83 to 1.80 g/L, provided that the NaNO_3_ concentration as kept below 6.0 g/L (Figures 1(b) and 1(c)).

Compared to the results of biomass concentration, the responses associated with lipid production were more bounded by the range of the input variables selected. Glucose of about 25 g/L was devoted to attain the maximum lipid yield and productivity at a given yeast extract (Figures 1(d) and 1(g)) and NaNO_3_ (Figures 1(e) and 1(h)) concentrations. Here, keeping the glucose at the center point would correspond to an optimal range of NaNO_3_ at 4.5 to 5.5 g/L, while yeast extract would be confined to a narrower range of 1.0 to 1.48 g/L (Figures 1(f) and 1(i)). Response surfaces with regard to lipid productivity depict an excellent circular contour, suggesting that the interaction between the input variables pose very little role in predicting the response [1]. Moreover, the quadratic terms were recognized to impart more influence towards regression modeling. Nonetheless, contour plots exhibiting a defined elliptical shape would otherwise indicate perfect interactions between the medium constituents used in formulation [14]. Such topology was visually evident in Figures 1(d) and 1(e). A cross referencing to ANOVA table confirms that the interaction between glucose and sodium nitrate (*x*_1_ *x*_3_) was actually very significant for directly promoting lipid yield in *Tetraselmis* cell (Prob > *F* = 0.0391). 

### 3.2. ANN Modeling

The overall lipid productivity was given more emphasis in algal cultivation. Biomass concentration on the other hand usually affects the downstream costs [11]. Thus, maximizing the main response of interest became the focal point of ANN optimization exercise. In the network training/testing process, a total of 330 neural network architectures were tested for the prediction of Pr_lipid_, each having a diverse configuration of hidden neurons, learning algorithm, and transfer functions of output and hidden layer. Nonetheless, it was necessary to ultimately choose only one of them, which provides the best compromise between bias and variance and also generates a good generalization.  Table 4 summarizes the top five ANN models.

Network training entails selecting a particular model that minimizes the error or cost criterion. Judging from Table 4, models with the least residual error were either trained using the Levenberg-Marquardt (LM) or Genetic algorithm (GA). LM is often regarded as the most efficient in terms of speed and accuracy in finding the optimal point compared to others [22]. Networks designed throughout this study were considered suitable to be trained by LM by abiding to the algorithm restrictions. Namely, LM is only effective for a small network (containing a few hundred weights) as its memory requirements are proportional to the square of the number of weights in the network, and the algorithm can only be used for network with a single output response. Additionally, LM is specifically used to minimize the sum of squares error and cannot be applied for other types of network errors. GA on the other hand is a stochastic method mostly associated with simulation of biologic heredities and evolutionary processes. Each possible solution to a set of problems is taken as an “individual” among population, and each individual is coded as a character string. GA applies its unique selection, crossing, and mutagenesis operators on a random population in order to compute a new one, eventually introducing some diversity to the algorithm [17]. An interesting trait of GA is that the algorithm is able to avoid a one-point optimal search usually associated with gradient descent or LM back propagation. Instead, GA is capable of global optimum exploration of the design space [23]. 

The choice of transfer function also directly affects the ANN's learning rate and is deemed instrumentally to its performance. In this study, most of the statistically accepted models were produced with linear function for output layer. Linear was frequently chosen for output layer for simulating functions without discontinuities. Gaussian, hyperbolic tangent, and sigmoid were all found to be suitable for hidden layer. Evidently from the tabulated results, the network using linear and sigmoid for the output and hidden layer produced the lowest RMSE (6.517) and a very high *R* (0.999) and DC (0.986). It has become a rule-of-thumb to choose sigmoid as the activation function for excellent non-linear model, but at the expense of slower learning [26]. Its hyperbolic tangent (Tanh) counterpart has the same response shape as that of sigmoid; thus, their computational cost is insignificantly different, and both functions can create a very smooth model. However, it was noted that the convergence performance of error functions was faster when Tanh function was employed for hidden layer. Calculated RMSE is slightly higher at 8.074, in addition to comparable *R* (0.995) and DC (0.978). Unlike sigmoid or Tanh function that acts as a gate (open or closed) for a neuron's output response when given a set of inputs, Gaussian behaves like a probabilistic output controller, producing output that can be described as a type of partial response. This transfer function tends to map pattern quicker than sigmoid; nevertheless, its prediction can be prone to memorization. 

**Figure 2:**
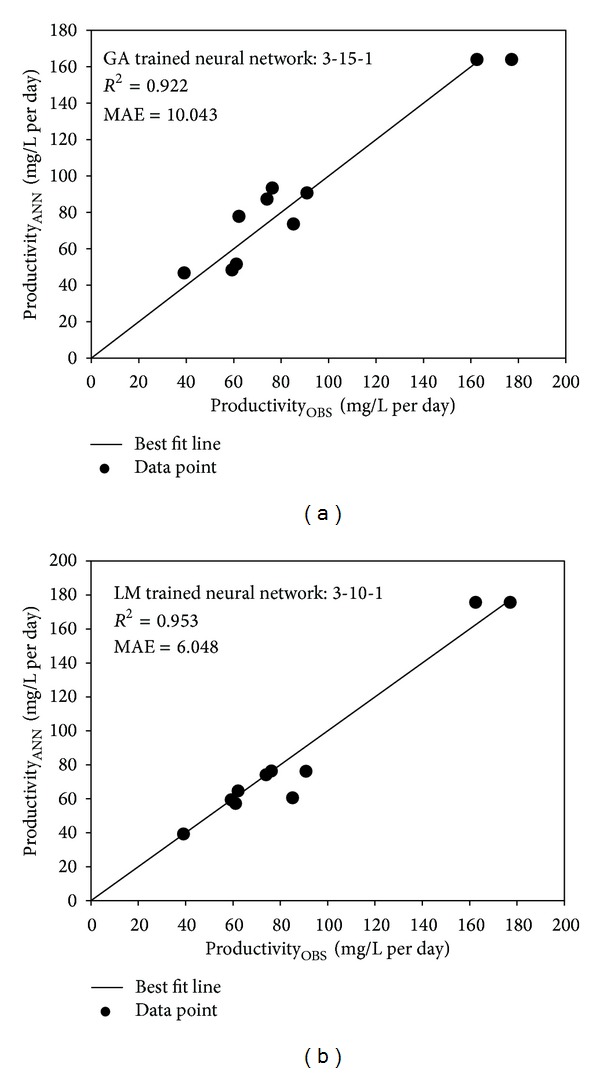
Parity plots correlating the observed and predicted values of the ANN models with respect to different testing dataset.

**Figure 3:**
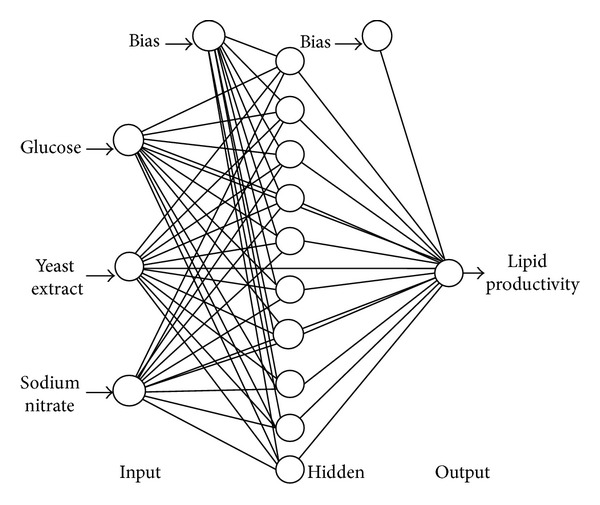
Finalized neural network architecture (3-10-1) trained via Levenberg-Marquardt algorithm for the estimation of lipid productivity.

The optimal number of neurons is an idiosyncrasy of the system in question. Increasing the neurons would rationally improve the learning performance, as too few would consequently lead to erratic learning or nonconvergence as observed in networks trained either with GA or LM. Network with too many neurons however may allow for too much freedom for the weights to adjust and, hence, invariably learn the noises that present in the training dataset [27]. To evaluate the fidelity of ANN architecture, parity plots of a testing set with 10 altogether different data points (Figure 2) were constructed for the top two networks in Table 4. The *R* ^2^ and MAE of the plots were then determined. Based on the fitting criteria, the LM-trained network of 3-10-1 architecture with Tanh function for hidden layer (Figure 3) was better in predicting the lipid productivity (*R* ^2^ of 0.953 and MAE of 6.048). Hence, the designed network could properly correlate the input and response. In most cases, good generalization could be obtained with ANN incorporating between 4 to 15 neurons [21].

**Figure 4:**
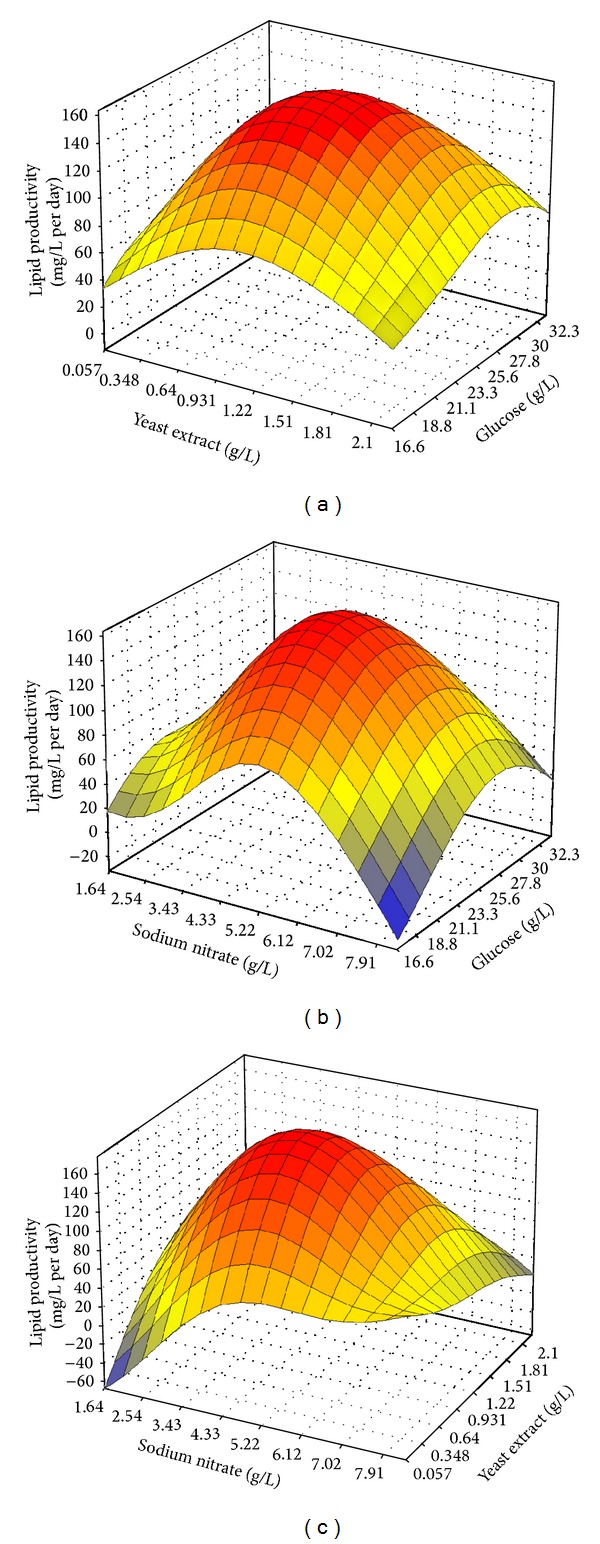
Response surfaces with regard to lipid productivity showing the interactions between (a) yeast extract with glucose, (b) NaNO_3_ with glucose, and (c) NaNO_3_ with yeast extract as modeled via neural network.

Figure 4 depicts the response surface topologies describing the interaction effect of the three medium constituents on lipid productivity as predicted by the optimal network. Every plot has a dome-shaped surface much similar to Figures 1(g) to 1(i). Notwithstanding, these plots project a distinctive undulated curvature, representing a graphical refinement in terms of nonlinearity in the output response compared with those generated by RSM.  Figure 4(a) shows that when NaNO_3_ is fixed at the middle level (5.0 g/L), lipid productivity increases when yeast extract and glucose are ramped up to a certain level before decreasing thereafter with further addition of these components. Similar trend persists in interaction between NaNO_3_ and glucose (Figure 4(b)) and also for interaction between yeast extract and NaNO_3_ (Figure 4(c)). 

### 3.3. Comparison of Predictive Capacity between RSM and ANN Models

The predicted responses computed via RSM and ANN are presented in Table 5. Evaluation based on the models' coefficient of determination actually shows a satisfactory convergence between the predicted and actual lipid productivity values. Thus, both models can be considered to perform well in data fitting and offered stable responses. Yet, the *R* ^2^ of ANN is closer to 1.0, indicating a higher predictive ability and accuracy as compared to RSM. Furthermore, RSM produced about 36.95% deviation in RMSE and about twice the difference in MAE than the error functions calculated from ANN-based approach (RMSE = 4.176 and MAE = 2.256). 

### 3.4. Optimization Employing Best Predicted Points of RSM and ANN Models

In RSM, the finalized medium composition was searched using the Design Expert optimization module with the goal of achieving the maximum lipid productivity. A single set of simulated solution was proposed comprising of (g/L): glucose, 28.11; NaNO_3_, 5.09; and yeast extract, 1.20. Highest lipid productivity was estimated at 169.20 mg/L per day with a desirability of 0.862. Alternatively, ANN calculates the optimum composition by ways of “Rotation Inherit Optimization” (RIO), an evolutionary algorithm much in-line with Genetic Algorithm (GA) or Particle Swarm Algorithm (PSA) albeit with faster convergence, and it dispenses with customized parameters set by experimenter, with the sole exception of population size. RIO was utilized to improve the best point searches of the studied system. Population size was set to 10. By 18000 iterations, the maximum lipid productivity was predicted at 174.84 mg/L per day. The resulting theoretical composition consisted of (g/L): glucose, 24.05; NaNO_3_, 4.70; and yeast extract, 0.93. Validation set was carried out and the results are compiled in Table 6. Formulation using RSM (160.17 mg/L per day) and ANN (173.11 mg/L per day) saw an increase of 1.76-fold and 1.90-fold of lipid productivity compared to the previous nonstatistically optimized run, in which W-30 medium was added with 30 g/L glucose. 

 The final results were observed to be insignificantly different for the medium formulated using the two statistical approaches. Nevertheless, it may still come as a surprise to see that culture grown in medium with lesser concentrations of glucose, NaNO_3_, and yeast extract could provide higher biomass and lipid productivity. This could be explained from the standpoint that unlike any synthetic medium prepared with distilled water, a workable concentration range of medium constituents adopted in this study was actually limited by the inherent physicochemical properties of full-strength (undiluted) seawater itself. The major obstacle would be to fully dissolve the nutrient components while simultaneously maintaining a circum-neutral pH tolerable to *Tetraselmis *survival. It was found that a too high concentration of yeast extract tends to lower the pH, and an attempt of readjusting by alkaline buffer (0.5 M NaOH) may be tampered with the existing solutes equilibrium and promote the constituents to form complexes with seawater, resulting in precipitation. Elevated alkalinity increases the supersaturation level of calcium ions in seawater and hence, leads to formation of amorphous calcium carbonate. This phenomenon would be enhanced either by the lack of magnesium ions present in seawater, or if there was an increased in iron (Fe^3+^), initiating the precipitation of calcite or potentially forming the colloidal iron hydroxide [28]. Fe^3+^ is an abundant and naturally occurring element in yeast extract (~2% w/w). In addition, a very high concentration of organic complex nutrient would obviously darken the medium. This diminishes the photosynthesis ability of microalga by impeding light penetration into deeper part of liquid culture. Both factors were postulated to have a deleterious effect on *Tetraselmis *growth. 

### 3.5. The Importance of Medium Components

**Figure 5:**
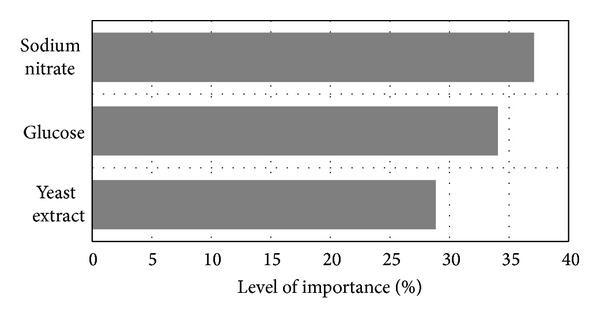
The level of importance of effective medium constituents on lipid productivity.

Figure 5 shows the degrees of importance (expressed in term of percentage of contribution) of the three medium constituents towards influencing lipid productivity as determined by NeuralPower. NaNO_3_ is the most important factor at 37.10%, followed by glucose at 34.06% and yeast extract at 28.84%. In general, nitrate is a major N source that strongly impacted the metabolism and growth of plant system. To assimilate nitrate, microalgae cells need to transport the ion across the membrane and subsequently reduce it to ammonia. The process is said to consume large amounts of energy, carbon, and protons [9]. Glucose is nonetheless a good choice for both carbon and energy sources for microalgae, since it can be easily stored as starch without prior conversion to glyceraldehyde phosphate (GAP) in Calvin's cycle. Plus, some part of it is readily oxidized throughout the glycolytic pathway [3]. The cheap and easily available yeast extract on the other hand was specifically chosen as it represents an economically sound and sustainable alternative for amino acids and vitamins sources.

**Figure 6:**
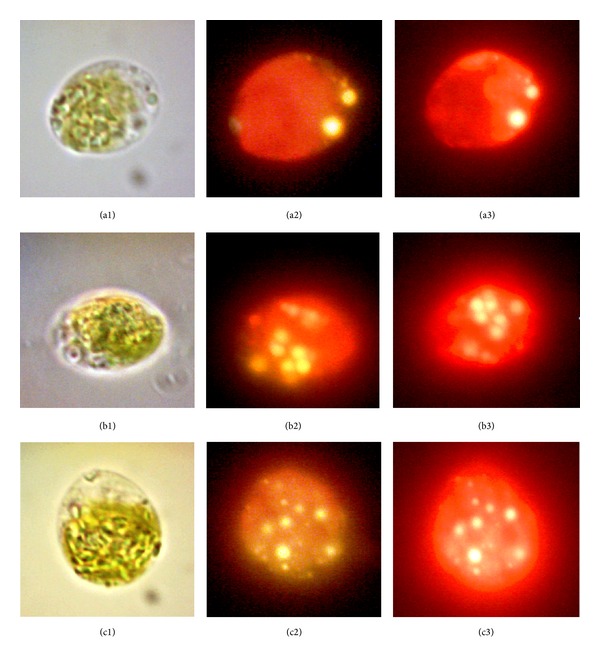
Photomicrographs of 1000x (1) phase contrast, (2) fluorescence of Nile red stained microalga viewed under excitation filter of 355–425 nm and emission filter of 470 nm, and (3) alternatively, excitation filter of 515–560 nm and emission filter of 590 nm of the 2-week old cultures. Red color, chlorophyll autofluorescence; yellow-gold fluorescence (excitation: 355–425 nm) or bright yellow fluorescence (excitation: 515–560 nm), lipid bodies. Samples were cultivated using (a) Walne's photoautotrophic medium, (b) W-30 + 30 g/L glucose, and (c) ANN-based optimized medium.

 Nile Red staining (Figure 6) has revealed the presence of substantial lipid globules inside *Tetraselmis* cells fed with glucose, in contrast with microalga grown under strict photoautotrophic after two-week cultivation. Introduction of organic carbon source and medium optimization had therefore yielded cultures with oil content consistent with the upper range reported for *Tetraselmis *species [2] in conjunction with rapid growth and higher biomass concentration. In microalgae cell, starch and lipid biosynthesis are two competing pathways of reduced carbon storage sink, whereby starch usually dominates over lipid under normal condition. Recent metabolic study has suggested that the high production rate of triacylglyceride (neutral lipid) would take place whenever the carbon supply exceeds the cell's capacity for starch synthesis in algal system. Using *Chlamydomonas reinhardtii *as model microalga, Fan et al. [29] have reported that feeding acetate at several-fold the concentration of the standard growth medium, a strategy termed as “mega-dosing,” would max out the cellular starch production capacity to the point that any additional carbon would be channeled into high-gear oil production. However, it should be noted that the substrate should not exceed the growth inhibitory level of the particular algal species. In essence, carbon precursor availability, as well as the notion of N starvation, is now accepted as the key metabolic factors controlling the partitioning of carbon into lipid in mixotrophic cultivation.

## 4. Conclusions

This study has shown that statistical techniques such as RSM and ANN could predict the *Tetraselmis'* biomass and intracellular lipid productivity. Though ANN may appear to be superior in terms of accuracy over RSM, it is opined here that both methodologies complemented each other in interpreting the results, whether in pointing out synergistic interactions among the input variables via ANOVA, or in classifying the importance of each component. ANN is unrestricted to the order of the model, and therefore, the approach is more dynamic in simulating the true behavior of nonlinear dataset. However, the typical downside of ANN requiring large amounts of training data for pattern recognition was circumvented through CCD initially devised for RSM. CCD is known to be an efficient design-of-experiment method with a hypercube geometry region, which is the best for minimizing the number of runs while upholding statistical significance. Addition of yeast extract into W-30 medium composition significantly enhanced the algal growth to as much as 12.38 g/L, but did not increase the proportion of lipid bodies (195.77 mg/g dcw) higher than the maximum reported in the literature.

## Figures and Tables

**Table 1:** Actual levels for independent variables (medium components) designed through CCD approach.

Factors	Symbols	Coded levels				
		−1.6818	−1	0	+1	+1.6818
Glucose (g/L)	*x*_1_	16.59	20	25	30	33.50
Yeast extract (g/L)	*x*_2_	0.06	0.50	1.15	1.80	2.25
Sodium Nitrate (g/L)	*x*_3_	1.65	3	5	7	8.35

**Table 2:** Central composite design (CCD) used in RSM and ANN studies showing the actual levels and observed responses during the mixotrophic cultivation *Tetraselmis *sp. FTC 209.

Run	*x*_1_	*x*_2_	*x*_3_	*X*_max⁡_ (g/L)	Lipid yield, *P*_max⁡_/*X*_max⁡_ (mg/g dcw)	Lipid productivity, Pr_lipid_ (mg/L per day)
1	20.00	0.50	3.00	4.955	76.81	31.72
2	30.00	0.50	3.00	8.15	87.36	59.33
3	20.00	1.80	3.00	9.325	75.60	58.75
4	30.00	1.80	3.00	9.65	77.99	62.72
5	20.00	0.50	7.00	7.365	98.30	60.33
6	**30.00**	**0.50**	**7.00**	**10.1**	**72.67**	**61.17**
7	20.00	1.80	7.00	7.245	105.31	63.58
8	**30.00**	**1.80**	**7.00**	**12.69**	**58.87**	**62.26**
9	16.59	1.15	5.00	6.835	130.03	74.06
10	33.50	1.15	5.00	13.05	70.25	76.40
11	25.00	0.06	5.00	8.6	85.28	61.12
12	25.00	2.25	5.00	10.195	61.11	56.00
13	**25.00**	**1.15**	**1.65**	**4.315**	**108.95**	**39.18**
14	25.00	1.15	8.35	6.275	81.17	42.44
15	**25.00**	**1.15**	**5.00**	**11.555**	**168.83**	**162.57**
16	25.00	1.15	5.00	10.175	204.29	173.22
17	25.00	1.15	5.00	12	177.18	177.18
18	25.00	1.15	5.00	11.07	171.40	158.11
19	25.00	1.15	5.00	11.55	183.30	176.42

ANN training set: normal numbers; ANN testing set: bold numbers.

**Table 3:** ANOVA tables showing the regression model on ^a^biomass concentration, ^b^lipid yield, and ^c^lipid productivity.

Source	Sum of squares	DF	Mean squares	*F* value	Prob > *F*	
Biomass concentration^a^						
Model	115.55	9	12.84	14.20	0.0003	Significant
A (glucose)	37.52	1	37.52	41.49	0.0001	
B (yeast extract)	12.12	1	12.12	13.41	0.0052	
C (NaNO_3_)	6.09	1	6.09	6.73	0.0290	
A^2^	1.91	1	1.91	2.11	0.1804	
B^2^	2.45	1	2.45	2.70	0.1345	
C^2^	55.43	1	55.43	61.30	<0.0001	
AB	0.05	1	0.05	0.06	0.8117	
AC⁡	2.16	1	2.16	2.39	0.1563	
BC	1.90	1	1.90	2.10	0.1810	
Residual	8.14	9	0.90			
Lack of Fit	6.21	5	1.24	2.57	0.1907	Not significant
Pure Error	1.93	4	0.48			
Cor Total	123.69	18				

Lipid yield^b^						
Model	38640.62	9	4293.40	22.14	<0.0001	Significant
A (glucose)	1454.22	1	1454.22	7.50	0.0229	
B (yeast extract)	168.75	1	168.75	0.87	0.3752	
C (NaNO_3_)	27.36	1	27.36	0.14	0.7159	
A^2^	12340.99	1	12340.99	63.65	<0.0001	
B^2^	20415.60	1	20415.60	105.30	<0.0001	
C^2^	13070.60	1	13070.60	67.41	<0.0001	
AB	189.90	1	189.90	0.98	0.3482	
AC⁡	1128.46	1	1128.46	5.82	0.0391	
BC	4.83	1	4.83	0.02	0.8781	
Residual	1744.99	9	193.89			
Lack of Fit	942.45	5	188.49	0.94	0.5391	Not significant
Pure Error	802.53	4	200.63			
Cor Total	40385.61	18				

Lipid productivity^c^						
Model	48411.82	9	5379.09	64.82	<0.0001	Significant
A (glucose)	160.43	1	160.43	1.93	0.1978	
B (yeast extract)	80.03	1	80.03	0.96	0.3518	
C (NaNO_3_)	163.34	1	163.34	1.97	0.1942	
A^2^	14549.79	1	14549.79	175.32	<0.0001	
B^2^	19719.16	1	19719.16	237.61	<0.0001	
C^2^	26767.04	1	26767.04	322.53	<0.0001	
AB	133.84	1	133.84	1.61	0.2360	
AC⁡	190.05	1	190.05	2.29	0.1645	
BC	136.06	1	136.06	1.64	0.2324	
Residual	746.92	9	82.99			
Lack of Fit	448.49	5	89.70	1.20	0.4416	Not significant
Pure Error	298.43	4	74.61			
Cor Total	49158.74	18				

**Table 4:** The effect of different full feed-forward network architectures on the model residual error (RMSE, *R*, and DC) in the prediction of lipid productivity from mixotrophic *Tetraselmis* sp.

Set	Model	Learning algorithm	Transfer function hidden	Transfer function output	Training set RMSE	Training set *R*	Training set DC	Testing set RMSE	Testing set *R*	Testing set DC
1	3-15-1	GA	SIGMOID	LIN	4.122	0.996	0.993	6.517	0.999	0.986
2	3-10-1	LM	TANH	LIN	4.122	0.996	0.993	8.074	0.995	0.978
3	3-15-1	LM	TANH	LIN	4.126	0.996	0.993	9.790	0.991	0.968
4	3-9-1	GA	GAUSS	LIN	4.126	0.996	0.993	10.294	0.992	0.965
5	3-10-1	GA	GAUSS	LIN	4.126	0.996	0.993	13.033	0.999	0.947

**Table 5:** Predicted lipid productivity by RSM and ANN together with the residual error functions (*R* ^2^, RMSE, and MAE).

Run	RSM predicted lipid productivity	RSM absolute deviation	ANN predicted lipid productivity	ANN absolute deviation
1	37.150	5.430	31.715	0.005
2	56.735	2.595	59.333	0.003
3	53.952	4.848	58.750	0.050
4	60.632	2.088	62.719	0.001
5	57.594	2.736	60.333	0.003
6	61.140	0.030	61.473	0.303
7	61.356	2.224	63.583	0.003
8	53.997	8.263	62.356	0.096
9	74.311	0.251	74.063	0.002
10	82.909	6.509	74.961	1.439
11	58.749	2.371	61.760	0.640
12	65.189	9.189	54.854	1.146
13	39.258	0.078	35.486	3.694
14	49.187	6.747	43.021	0.581
15	169.466	6.696	171.234	8.664
16	169.466	3.954	171.234	1.986
17	169.466	7.914	171.234	5.946
18	169.466	11.156	171.234	13.124
19	169.466	7.154	171.234	5.186

RSM Model *R* ^2^ = 0.985, ANN testing set *R* ^2^ = 0.993.

RSM Model RMSE = 5.719, ANN testing set RMSE = 4.176.

RSM Model MAE = 4.750, ANN testing set MAE = 2.256.

**Table 6:** Validation results of medium compositions as suggested by RSM and ANN models.

*Tetraselmis* sp. FTC 209 cultivation performance	Optimum points prediction		W-30 mixotrophic	Walne's autotrophic
	RSM	ANN		
Glucose (g/L)	28.11	24.05	30	—
Sodium Nitrate (g/L)	5.10	4.70	5.24	0.10
Yeast Extract (g/L)	1.20	0.93	—	—
Predicted Pr_lipid_ (mg/L per day)	169.20	175.84	—	—
Observed Pr_lipid_ (mg/L per day)	160.17	173.11	90.90	4.82
*X*_max⁡_ (g/L)	11.86	12.38	8.10	0.830
*P*_max⁡_/*X*_max⁡_ (mg/g dcw)	189.07	195.77	224.78	122.30

## References

[1] Azma M, Mohamed MS, Mohamad R, Rahim RA, Ariff AB (2011). Improvement of medium composition for heterotrophic cultivation of green microalgae, *Tetraselmis suecica*, using response surface methodology. *Biochemical Engineering Journal*.

[2] Chisti Y (2007). Biodiesel from microalgae. *Biotechnology Advances*.

[3] Huang G, Chen F, Wei D, Zhang X, Chen G (2010). Biodiesel production by microalgal biotechnology. *Applied Energy*.

[4] Gladue RM, Maxey JE (1994). Microalgal feeds for aquaculture. *Journal of Applied Phycology*.

[5] Bumbak F, Cook S, Zachleder V, Hauser S, Kovar K (2011). Best practices in heterotrophic high-cell-density microalgal processes: achievements, potential and possible limitations. *Applied Microbiology and Biotechnology*.

[6] Day JG, Tsavalos AJ (1996). An investigation of the heterotrophic culture of the green alga *Tetraselmis*. *Journal of Applied Phycology*.

[7] De la Hoz Siegler H, Ben-Zvi A, Burrell RE, Mccaffrey WC (2011). The dynamics of heterotrophic algal cultures. *Bioresource Technology*.

[8] Wu X, Ruan R, Du Z, Liu Y (2012). Current status and prospects of biodiesel production from microalgae. *Energies*.

[9] Perez-Garcia O, Escalante FME, de-Bashan LE, Bashan Y (2011). Heterotrophic cultures of microalgae: metabolism and potential products. *Water Research*.

[10] Zhao G, Yu J, Jiang F, Zhang X, Tan T (2012). The effect of different trophic modes on lipid accumulation of *Scenedesmus quadricauda*. *Bioresource Technology*.

[11] Isleten-Hosoglu M, Ayyildiz-Tamis D, Zengin G, Elibol M (2013). Enhanced growth and lipid accumulation by a new *Ettlia texensis* isolate under optimized photoheterotrophic condition. *Bioresource Technology*.

[12] Danquah MK, Harun R, Halim R, Forde GM (2010). Cultivation medium design via elemental balancing for *Tetraselmis suecica*. *Chemical and Biochemical Engineering Quarterly*.

[13] Mandal S, Mallick N (2009). Microalga *Scenedesmus obliquus* as a potential source for biodiesel production. *Applied Microbiology and Biotechnology*.

[14] Xie T, Sun Y, Du K, Liang B, Cheng R, Zhang Y (2012). Optimization of heterotrophic cultivation of *Chlorella* sp. for oil production. *Bioresource Technology*.

[15] Wu Z, Shi X (2007). Optimization for high-density cultivation of heterotrophic *Chlorella* based on a hybrid neural network model. *Letters in Applied Microbiology*.

[16] Karim MN, Hodge D, Simon L (2003). Data-based modeling and analysis of bioprocesses: some real experiences. *Biotechnology Progress*.

[17] García-Gimeno R, Hervás-Martíanez C, Barco-Alcalá E, Zurera-Cosano G, Sanz-Tapia E (2003). An artificial neural network approach to *Escherichia coli* O157:H7 growth estimation. *Journal of Food Science*.

[18] Razmi-Rad E, Ghanbarzadeh B, Rashmekarim J (2008). An artificial neural network for prediction of zeleny sedimentation volume of wheat flour. *International Journal of Agriculture and Biology*.

[19] Cid A, Abalde J, Herrero C (1992). High yield mixotrophic cultures of the marine microalga *Tetraselmis suecica* (Kylin) Butcher (Prasinophyceae). *Journal of Applied Phycology*.

[20] Ferreira IMPLVO, Pinho O, Vieira E, Tavarela JG (2010). Brewer’s Saccharomyces yeast biomass: characteristics and potential applications. *Trends in Food Science and Technology*.

[21] Tan JS, Ramanan RN, Ling TC, Shuhaimi M, Ariff AB (2010). Comparison of predictive capabilities of response surface methodology and artificial neural network for optimization of periplasmic interferon-*α*2b production by recombinant *Escherichia coli*. *Minerva Biotecnologica*.

[22] Moghaddam MG, Ahmad FBH, Basri M, Rahman MBA (2010). Artificial neural network modeling studies to predict the yield of enzymatic synthesis of betulinic acid ester. *Electronic Journal of Biotechnology*.

[23] Ghaffari A, Abdollahi H, Khoshayand MR, Bozchalooi IS, Dadgar A, Rafiee-Tehrani M (2006). Performance comparison of neural network training algorithms in modeling of bimodal drug delivery. *International Journal of Pharmaceutics*.

[24] Folch J, Folch M, Sloane-Stanley GH (1957). A simple method for the isolation and purification of total lipides from animal tissues. *The Journal of Biological Chemistry*.

[25] Chen W, Zhang C, Song L, Sommerfeld M, Hu Q (2009). A high throughput Nile red method for quantitative measurement of neutral lipids in microalgae. *Journal of Microbiological Methods*.

[26] Duch W, Jankowski N (1999). Survey of neural transfer functions. *Neural Computing Surveys*.

[27] Basri M, Rahman RNZRA, Ebrahimpour A, Salleh AB, Gunawan ER, Rahman MBA (2007). Comparison of estimation capabilities of response surface methodology (RSM) with artificial neural network (ANN) in lipase-catalyzed synthesis of palm-based wax ester. *BMC Biotechnology*.

[28] Charles BM, Patricia AW (2012). *Biological Oceanography*.

[29] Fan J, Yan C, Andre C, Shanklin J, Schwender J, Xu C (2012). Oil accumulation is controlled by carbon precursor supply for fatty acid synthesis in *Chlamydomonas reinhardtii*. *Plant and Cell Physiology*.

